# Mycophenolate mofetil ameliorates diabetic nephropathy through epithelial mesenchymal transition in rats

**DOI:** 10.3892/mmr.2015.3934

**Published:** 2015-06-15

**Authors:** XIAOYAN XIAO, JIE WANG, XIANGDI CHANG, JUNHUI ZHEN, GENGYIN ZHOU, ZHAO HU

**Affiliations:** 1 Department of Nephrology, Qilu Hospital of Shandong University, Jinan, Shandong 250012, P.R. China; 2 Department of Nephrology, Liaocheng People's Hospital, Liaocheng, Shandong 252000, P.R. China; 3 Department of Nephrology, Affiliated Hospital of Binzhou Medical School, Binzhou Shandong 256603, P.R. China; 4 Department of Pathology, Qilu Hospital of Shandong University, Jinan, Shandong 250012, P.R. China

**Keywords:** benazepril, diabetic nephropathy, epithelial mesenchymal transition, mycophenolate mofetil, rats

## Abstract

Recent studies in animal models have revealed that mycophenolate mofetil (MMF) has certain protective effects against experimental diabetic nephropathy. The present study therefore aimed to investigate the hypothesis that diabetic nephropathy may be ameliorated by mycophenolate mofetil and benazepril treatment alone or in combination, and identify the potential underlying mechanisms in a rat model. Diabetes was induced in rats by a single intraperitoneal injection of streptozotocin. Rats were subsequently treated with benazepril, MMF or a combination of the two drugs, and blood glucose, normalized kidney weight, urine protein and serum creatinine were determined. The pathological changes in renal tissue were also observed. In addition, indices of epithelial mesenchymal transition, including α-smooth muscle actin (α-SMA) and transforming growth factor (TGF)-β_1_ expression, were examined. Normalized kidney weight, urine protein and serum creatinine levels were significantly improved in the diabetic rats treated with benazepril or mycophenolate mofetil, compared with those of rats in the untreated diabetic group. Pathological changes in the kidney were detected concurrently with increasing kidney weight and urinary albumin excretion, with a similar trend in variation among groups. In addition, the expression of epithelial mesenchymal transition indices, including α-SMA and TGF-β_1_, in the renal tubule interstitium were significantly decreased in the benazepril- and MMF-treated groups compared with those of the diabetic group. As expected, the aforementioned indices were markedly lower in the benazepril and MMF combined treatment group than those in the single medication groups. These data suggested that MMF may have a protective role in diabetic nephropathy, and that the underlying mechanism may be partially dependent upon the suppression of the epithelial mesenchymal transition. Furthermore, the combination of benazepril and MMF conferred enhanced efficacy over monotherapies in the treatment of diabetic nephropathy.

## Introduction

Diabetic nephropathy (DN) is one of the most common micro-vascular complications of diabetes, and is the leading cause of end stage renal diseases worldwide (1). The majority of past studies on DN have concentrated on the glomerular lesions (2). Conversely, the mechanism underlying the development of renal tubule interstitial lesions has been neglected; although the tubule interstitium occupies the majority of the total kidney volume. The significance of renal interstitial fibrosis (RIF) in the progression of DN has gained increasing attention. Numerous *in vivo* and *in vitro* studies have verified that the epithelial mesenchymal transition (EMT) of renal tubular epithelial cells is a key mechanism underlying RIF (3,4). EMT has been identified as a key contributor to the loss of renal function throughout the nephron in DN (5). Transforming growth factor (TGF)-β_1_ is a pro-sclerotic cytokine, which is associated with the EMT (6). The upregulation of TGF-β_1_ expression in diabetes identifies this pro-fibrotic cytokine as a potential candidate for mediation of the development of such fibrotic complications (5). TGF-β_1_-induced EMT is characterized by the loss of E-cadherin, cell adhesion and connexin-mediated cell communication in the proximal tubule under diabetic conditions; changes which occur prior to the development of overt signs of renal damage (7).

It has previously been demonstrated that the immunosuppressant mycophenolate mofetil (MMF) has certain protective effects in experimental DN, and that its mechanism may be associated with the inhibition of renal infiltrating inflammatory cells (8,9). Angiotensin converting enzyme inhibitors (ACEI) or angiotensin receptor antagonists may protect the kidney by reducing diabetic glomerular hypertension, as well as decreasing urinary protein and non-hemodynamic mechanisms. Renin angiotensin system (RAS) blocking, combined with MMF treatment, exerted markedly improved renal protection in a residual renal kidney model compared with MMF or RAS blocking monotherapy (10). However, whether MMF has specific protective effects against diabetic nephropathy and its underlying mechanism of action have remained to be elucidated.

In the present study, diabetic rats were treated with benazepril (a representative ACEI) and/or MMF and the role of MMF in DN was investigated using a streptozocin-induced diabetic Sprague-Dawley rat model.

## Materials and methods

### Experimental animals

Forty adult male Sprague-Dawley rats (180–200 g) were purchased from Beijing Vital River Laboratory Animal Technology Co., Ltd (Beijing, China) and maintained in the animal facility of Qilu Hospital of Shandong University (Jinan, China). All rats were housed under controlled temperature (22±1°C), humidity (65–70%) and lighting (12/12 h circadian cycle), with *ad libitum* access to standard rat diet and sterile water throughout the study. All animal experimental procedures were performed in accordance with the Guidelines for Animal Experiments of Qilu Hospital of Shandong University and were approved by the Institutional Ethics Committee for Laboratory Animal Care of Qilu Hospital, Shandong University.

### Determination of diabetes and diabetic nephropathy

Following one week of feeding, eight rats were randomly selected to be the normal control group (group N), and the remaining 32 rats were administered a single intraperitoneal injection of streptozotocin (STZ; 60 mg/kg) (Sigma-Aldrich, St. Louis, MO, USA) with 0.1 mmol/l citric acid as buffer (Fuzhou Maixin Biotech. Co., Ltd., Fuzhou, China). The control rats received an injection of an identical volume of citric acid buffer alone. Seventy-two hours following injection, blood glucose (BG) was detected each day, for the subsequent three days. Diabetic rats were administered a sub-therapeutic dose of insulin (Eli Lilly, Indianapolis, IN, USA) following diagnosis, in order to maintain the animals in a hyperglycemic state, ranging between 13.9 and 22.2 mmol/l, however in relatively good general health. Diabetes induction was confirmed by three consecutive readings of BG>16.7 mmol/l (300 mg/dl).

### Experimental groups and treatment

The diabetic rats were randomly divided into four groups: The diabetes mellitus (DM) group, received no treatment (n=8); B group, treated with benazepril (Beijing Nuohua China Pharmaceutical Co., Beijing, China; n=8); M group, treated with MMF (Roche Pharmaceutical Co., Basel, Switzerland; n=8) and the BM group (n=8) treated with benazepril and MMF. Rats were treated with drugs (10 mg/kg) daily by gavage administration for eight weeks, beginning four weeks following successful confirmation of the diabetic model, and the corresponding normal controls were given identical volumes of distilled water. BG and proteinuria were monitored weekly. Rats were anesthetized by intraperitoneal injection of 10% chloral hydrate (3 ml/kg) on week 12. Blood was obtained through the inferior vena cava following anesthesia and centrifuged at 1,300 × g for 10 min. Serum was stored at −80°C for biochemical indices measurement. Pre-cooled normal saline was injected into the rat left ventricle and the right atrial was incised for drainage. Kidneys were repeatedly lavaged until the entire kidney turned pale. Kidneys were removed, weighed following suck dry of filter paper. One kidney was cut along through the hilum in the coronal plane. One half of the kidney was fixed in 10% neutral formalin and reserved for pathological examination and immunohistochemical specimens; the other half was immersed in fresh fixatives for electron microscopy. The remaining kidney was snap frozen in liquid nitrogen and stored at −80°C for western blot analysis.

### Urine albumin and creatinine measurements

Twenty-four hour urine collection measurements were obtained using metabolic cages, and urine albumin was quantified using a protein assay kit purchased from Bio-Rad Laboratories, Inc. (Hercules, CA, USA). Urine creatinine was measured using a Cayman creatinine assay kit (Ann Arbor, MI, USA).

### Histology

Kidneys were perfused with ice-cold phosphate-buffered saline (PBS), fixed in 10% buffered formaldehyde for two days, embedded in paraffin (Fuzhou Maixin Biotech. Co., Ltd.) and processed for sectioning. The sections were 5 *µ*m thick and ~30 sections were obtained from each kidney. These sections were used for hematoxylin and eosin, periodic acid/Schiff (PAS), Masson, α-SMA and TGF-β1 immunohistochemical staining. Pathological changes were detected by hematoxylin and eosin staining (Fuzhou Maixin Biotech. Co., Ltd.), observed using light microscopy (Nikon 80i; Nikon, Tokyo, Japan). Extracellular matrix deposition (mesangial matrix expansion) in the glomeruli was assessed with PAS staining (Fuzhou Maixin Biotech. Co., Ltd.). Collagen fibers were stained by Masson staining (Fuzhou Maixin Biotech. Co., Ltd.).

### PAS staining

Paraffin sections were deparaffinized, oxidated in 1% aqueous periodate solution for 15 min and washed three times in distilled water. Sections were then soaked in Schiff fluid for 10–30 min (control time under a microscope), washed and dyed with hematoxylin for 2 min. Sections were differentiated by 1% hydrochloric acid alcohol, rinsed with water until the nucleus turned blue and dehydrated by gradient alcohol and mounted with resinous mounting medium. The PAS positive reaction was red and the nuclei were blue under the microscope (Nikon 80i; Nikon, Tokyo, Japan).

### Masson staining

Paraffin sections were deparaffinized and dyed with hematoxylin for 10 min. Sections were rinsed in warm tap water for 10 min, washed in distilled water and stained in Biebrich scarlet-acid fuchsin solution for 10–15 min. Following washing in distilled water, sections were differentiated in phosphomolybdic-phosphotungstic acid solution for 10–15 min or until the collagen was not red. Sections were directly transferred to aniline blue solution and stained for 5–10 min. Following rinsing briefly in distilled water, sections were differentiated in 1% acetic acid solution for 2–5 min, washed in distilled water and dehydrated quickly through 95% ethyl alcohol, absolute ethyl alcohol until they were mounted with resinous mounting medium. The collagen was blue, nuclei were black and the cytoplasm was red under the microscope (Nikon 80i; Nikon, Tokyo, Japan).

### Determination of tubulointerstitial injury index

Twenty high-power fields (magnification, x200) per section for each sample with PAS staining were randomly selected and evaluated by three pathologists. The tubulointerstitial injury index was scored as the percentage of tubulointerstitial injury area within the total area: 0, normal; 1, <25%; 2, 25–50%; 3, >50% (11).

### Immunohistochemistry

Specimens were deparaffinized and rehydrated with xylene and 80, 90, 95 and 100% ethanol. Following washing three times with PBS, sections were placed in 0.01 mol/l citrate buffer (pH 6.0), heated in a microwave (92–95°C, 15 min) for antigen retrieval and cooled to room temperature. Following antigen retrieval, primary antibodies, including mouse anti-rat α-smooth muscle actin (α-SMA) monoclonal antibody (Abcam, Cambridge, UK; cat. no. ab7817) and mouse anti rat TGF-β_1_ monoclonal antibody (Abcam; cat. no. ab64715) were used at dilutions of 1:75 and 1:50, respectively, and incubated overnight at 4°C. Immunoglobulin G-conjugated horseradish peroxidase (HRP) goat anti-mouse polyclonal antibody (1:500; cat. no. ZB-2305; Beijing ZSGB Biotech. Co., Ltd., Beijing, China) and 3,3-diaminobenzidine tetrahydrochloride (Vector Laboratories, Burlingame, CA, USA) were employed to visualize antibody binding (control time under a microscope at room temperature). Positive staining was defined as the presence of tan-yellow color granular staining. Optical density values of positive areas in each slice were calculated using Image-Pro Plus 6.0 analysis software (Media Cybernetics, Inc., Rockville, MD, USA).

### Electron microscopy

Following perfusion, the kidneys were excised and immersed in fresh fixative comprised of 2.5% glutaraldehyde in 0.1 M Na cacodylate buffer (pH 7.4; Fuzhou Maixin Biotech. Co., Ltd.) for ~16 h at 4°C. The tissue blocks were post-fixed with 1% osmium tetroxide/0.8% potassium ferricyanide in 0.1 M cacodylate buffer, treated with aqueous 1% uranyl acetate, dehydrated in graded ethanol solutions (Fuzhou Maixin Biotech. Co., Ltd.) and embedded in Polybed epoxy resin for morphological analysis (Polysciences, Warrington, PA, USA). Sections were cut (70 nm), placed on 200-mesh copper/rhodium grids and stained with uranyl acetate and lead citrate (Fuzhou Maixin Biotech. Co., Ltd.), prior to observation at 60 kV on a Philips CM10 transmission electron microscope (Philips, Eindhoven, The Netherlands). The thickness of the basal membrane was measured using a validated simplified method (12).

### Western blot analysis

Kidney tissues were resuspended in ice-cold radioimmunoprecipitation assay buffer (Bio-Rad Laboratories, Inc.) for lysis. For the protein assay, a bicinchoninic acid protein assay kit (Beyotime Institute of Biotechnology, Haimen, China) was used. Approximately 30 *µ*g protein samples were loaded, separated on 9.0% Tris-Glycine-SDS-PAGE and electroblotted onto polyvinylidene difluoride membranes (Bio-Rad Laboratories, Inc.). The membranes were then incubated for 1 h in a blocking solution containing 5% non-fat milk in a PBS 0.05% Tween solution. Following blocking, membranes were incubated with the mouse anti-rat α-SMA monoclonal antibody (1:250) or the mouse anti rat TGF-β_1_ monoclonal antibody (1:500; Abcam) overnight at 4°C. Membranes were subsequently incubated with polyclonal goat anti-mouse HRP-conjugated secondary antibody (Santa Cruz Biotechnology, Inc., Dallas, TX, USA) for 1 h at 37°C. Immunoreactive bands were detected using a chemiluminescence detection kit (Amersham Pharmacia Biotech, Piscataway, NJ, USA). Band intensities were quantified using Image J 1.32 software (National Institutes of Health, Bethesda, MD, USA). β-actin was used as an internal loading control.

### Statistical analysis

Values are presented as the mean ± standard deviation. Statistical analysis was performed using GraphPad Prism software version 4.0 (GraphPad Software, Inc., San Diego, CA, USA). One-way analysis of variance was performed where appropriate. Post-hoc Bonferroni pair-wise comparisons were used to assess significant differences between two groups. P<0.05 was considered to indicate a statistically significant difference.

## Results

### Kidney hypertrophy, urinary albumin excretion and renal function are improved following MMF treatment in diabetic rats

**Figure 1:**
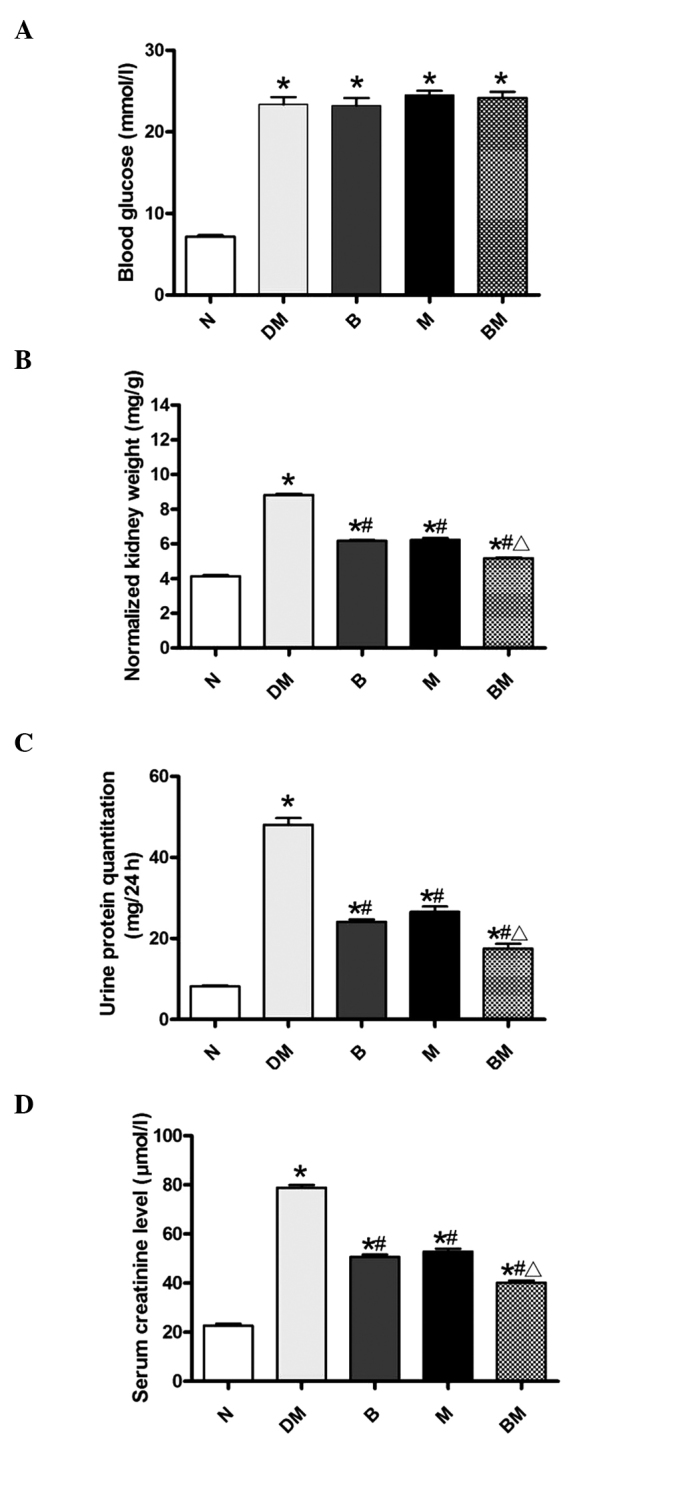
Changes in blood glucose, normalized kidney weight, urine protein and serum creatitine levels amongst the five groups. (A) Average blood glucose levels in diabetic rats (DM, B, M, BM) compared with non-diabetic rats (N). (B) Normalized kidney weight. (C) Urinary albumin excretion over 24 h. (D) Serum creatitine levels. Values are presented as the mean ± standard deviation. ^*^P<0.05, vs. N; ^#^P<0.05, vs. DM; ^Δ^P<0.05, vs. B or M; n=8 per group. N, non-diabetic control; DM, diabetic rats; B, diabetic rats treated with benazepril; M, diabetic rats treated with mycophenolate mofetil; BM, diabetic rats treated with benazepril and mycophenolate mofetil.

Following the successful induction of diabetes in the rat model, rats gradually developed polydipsia, polyuria, polyphagia, weight loss, grey hair and slow reactions in the DM group. Mean BG levels remained unchanged in the DM group with sub-therapeutic doses of insulin over the 12-week experimental period, although the BG levels were significantly higher than those in the non-diabetic control rats (Fig. 1A). There was no significant difference in the normalized kidney weight between groups at the commencement of the experiment; however, the normalized kidney weight had significantly increased in the DM group compared with that of the N group upon termination of the experiments, suggesting the presence of kidney hypertrophy (Fig. 1B). The normalized kidney weight increased in the benazepril, MMF and combination regimen groups; however, the kidney weight was significantly lower than that of the DM group. Notably, the increase in kidney weight was markedly smaller in the combination regimen group than that in the benazepril or MMF groups (Fig. 1B).

As expected, urinary albumin excretion and serum creatitine (Scr) revealed a similar trend to that observed with the normalized kidney weight amongst the groups (Fig. 1C and D). Twenty-four hour urinary protein and Scr levels were significantly increased in the DM group compared with those of the control group; however this increase was attenuated following benazepril or MMF treatment. A greater improvement in these two indices was observed in the combination regimen group compared with the single-drug treatment groups (Fig. 1C and D).

### MMF treatment decreases glomerular hypertrophy and mesangial matrix expansion in diabetic rats

**Figure 2:**
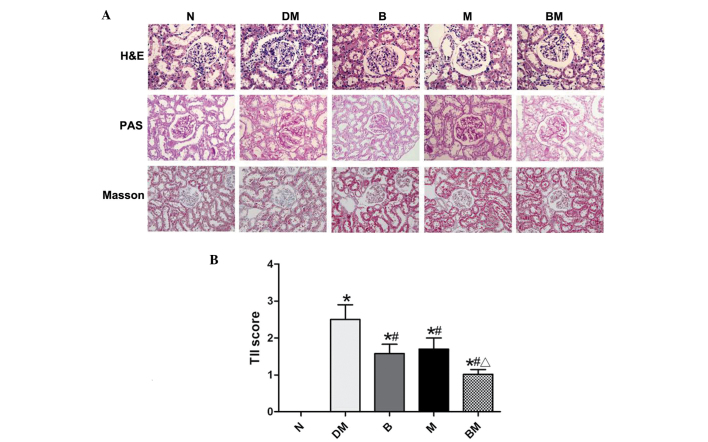
(A) Representative histology of renal sections stained with H&E, PAS and Masson in the various groups (magnification, x40). (B) TII scores. Values are presented as the mean ± standard deviation. ^*^P<0.05, vs. N; ^#^P<0.05, vs. DM; ^Δ^P<0.05, vs. B or M; n=8 per group. N, non-diabetic control; DM, diabetic rats; B, diabetic rats treated with benazepril; M, diabetic rats treated with mycophenolate mofetil; BM, diabetic rats treated with benazepril and mycophenolate mofetil; H&E, hematoxylin and eosin; PAS, periodic acid/Schiff; TII, tubular injury index.

Mesangial matrix expansion is one of the hallmarks of diabetic nephropathy, and was therefore investigated in diabetic rats in the present study via analysis of kidney sections obtained from the various groups. As indicated in Fig. 2, glomerular hypertrophy and accelerated mesangial matrix expansion, characterized by increased areas positive for PAS staining, were identified in the DM group compared with that of the non-diabetic controls. Glomerular hypertrophy and mesangial matrix expansion were attenuated following benazepril or MMF treatment. Notably, lesions in the BM group were markedly fewer than those in the single-drug groups. Masson staining additionally indicated increased levels of collagen in the DM group compared with those of the control group. However, collagen generation was ameliorated in the treatment groups (Fig. 2).

**Figure 3:**
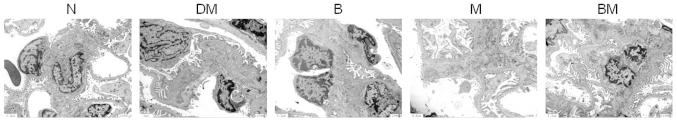
Electron micrographs of the glomeruli of the various groups (magnification, x15,000). N, non-diabetic control; DM, diabetic rats; B, diabetic rats treated with benazepril; M, diabetic rats treated with mycophenolate mofetil; BM, diabetic rats treated with benazepril and mycophenolate mofetil, n=2 per group.

In order to further investigate the structural alterations of the glomeruli, particularly those of the glomerular basement membrane (GBM), samples were examined using transmission electron microscopy. The GBM was found to be thicker in diabetic rats compared with that of the non-diabetic controls; however, these alterations in thickness were attenuated in the benazepril, MMF and combined treatment groups (Fig. 3). Extracellular matrix accumulation was also detected in the diabetic kidneys under electron microscopy, and this effect was also attenuated in the single and combined treatment groups (Fig. 3).

**Figure 4:**
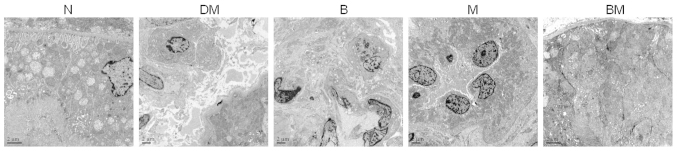
Electron micrographs of tubules in the various groups (magnification, x15,000). N, non-diabetic control; DM, diabetic rats; B, diabetic rats treated with benazepril; M, diabetic rats treated with mycophenolate mofetil; BM, diabetic rats treated with benazepril and mycophenolate mofetil. n=2 per group.

Renal tubule and interstitial lesions were also found to be more severe in the DM group than those present in the normal control and medicated groups. Renal tubular epithelial cell particle vacuole degeneration, renal tubular expansion and renal interstitial inflammatory cell infiltration were more marked in the DM group compared with those of the control group; while these lesions were significantly reduced in the benazepril, MMF and combined treatment groups (Fig. 4).

### EMT is involved in DN and is ameliorated following MMF treatment

**Figure 5:**
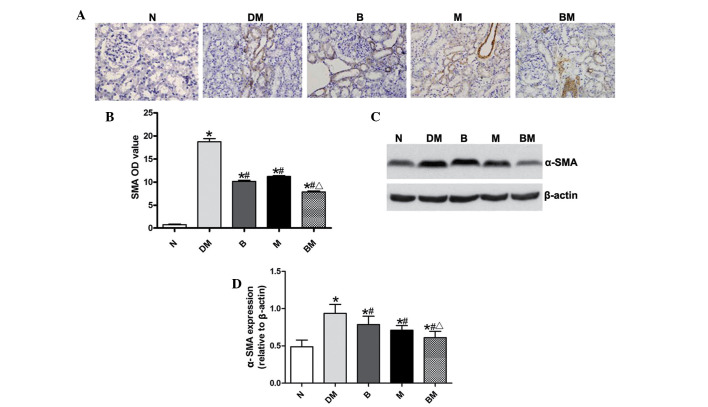
α-SMA expression in the five groups was analyzed. (A) Representative immunohistochemical staining of α-SMA expression in renal tissues of the five groups (magnification, x40). (B) OD values of α-SMA in the renal tubular interstitium were quantified. (C) Representative western blot analysis of α-SMA protein expression in renal tissues. (D) Relative quantitation of α-SMA protein. Data were obtained from three independent experiments in each condition. *P<0.05, vs. N; ^#^P<0.05, vs. DM; ^Δ^P<0.05, vs. B or M; n=8 for each group. N, non-diabetic control; DM, diabetic rats; B, diabetic rats treated with benazepril; M, diabetic rats treated with mycophenolate mofetil; BM, diabetic rats treated with benazepril and mycophenolate mofetil; α-SMA, α-smooth muscle actin.

To further evaluate whether EMT was involved in the development of DN, markers of EMT, including α-SMA and TGF-β_1_, were detected. As shown in Fig. 5A and B, α-SMA expression was only detected in the smooth muscle cells of the blood vessel walls in the normal control group. However, α-SMA expression was detected mainly in the renal tubular epithelial cell cytoplasm and tubule interstitium as well as in the walls of the blood vessels and surrounding the glomerulus in the DM group. Significantly increased α-SMA expression was detected in the DM group compared with that in the control group. Furthermore, α-SMA expression was significantly decreased in benazepril and MMF treatment groups compared with that in the DM group. In addition, α-SMA expression levels were markedly lower in the combined treatment group than those in the single-drug treatment groups. The results of western blot analysis of α-SMA protein expression were highly consistent with those observed by immunohistochemistry (Fig. 5C and D).

**Figure 6:**
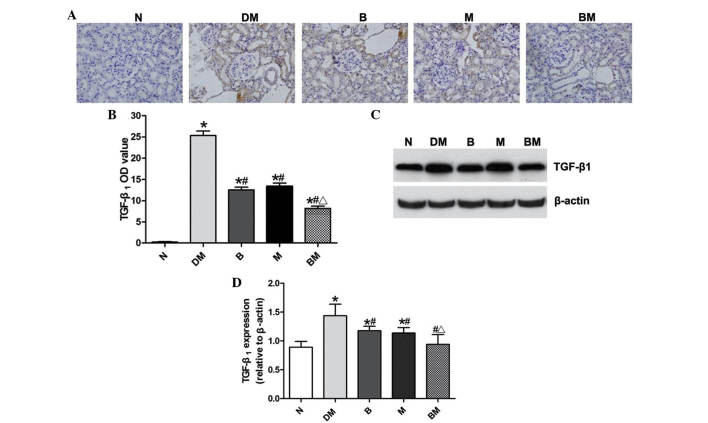
TGF-β_1_ expression was analyzed in the five groups. (A) Representative immunohistochemical staining of TGF-β_1_ expression in renal tissues of the five groups (magnification, x40). (B) OD values of TGF-β_1_ in renal tubular interstitium were quantified. (C) Representative western blot analysis of TGF-β_1_ protein in renal tissues. (D) Relative quantitation of TGF-β_1_ protein expression. Data were compiled from three independent experiments in each condition. *P <0.05 vs. N; ^#^P <0.05 vs. DM; ^Δ^P <0.05 vs. B or M; n=8 per group. N, non-diabetic control; DM, diabetic rats; B, diabetic rats treated with benazepril; M, diabetic rats treated with mycophenolate mofetil; BM, diabetic rats treated with benazepril and mycophenolate mofetil; TGF-β_1_, transforming growth factor-β_1_; OD, optical density.

In analogy with the trend observed in α-SMA expression, there was almost no TGF-β_1_ expression detected in the normal control group; whereas, TGF-β_1_ expression was significantly increased and mainly expressed in the renal tubular epithelial cell cytoplasm in the DM group (Fig. 6A and B). However, TGF-β_1_ expression was significantly decreased in the benazepril and MMF groups, compared with that of the DM group. In addition, TGF-β_1_ expression was significantly reduced in the combined treatment group, compared with the single drug treatment groups. Western blot analyses revealed analogous results to those identified by immunohistochemistry (Fig. 6C and D).

## Discussion

The results of the present study revealed that normalized kidney weight, 24 h urinary protein, renal function and renal pathological changes were markedly improved in the benazepril and MMF groups compared with those of the diabetic group at the end of the experimental period. Additionally, the improvement of these indices was greater in the combined benazepril and MMF treatment group compared with those in the single-drug treatment groups. Furthermore, there was no statistical difference in BG between the medicated groups and the DM group (Fig. 1A). All these results suggested that MMF has a protective role in DN, and that benazepril combined with MMF exerts coordinated protective effects against DN. Additionally, the protective effect of MMF was not dependent on hypoglycemic action.

Previous studies have demonstrated that tubulointerstitium pathological alterations occur at the same time or even prior to the appearance of alterations in the glomerular filtration membrane in DN (13), indicating that the development of renal tubular interstitial lesions is not entirely dependent on glomerular lesions, but is an independent factor associated with DN. It has been confirmed that the severity of tubulointerstitial lesions is closely associated with urinary protein excretion and the progressive decline in renal function, as well as directly influencing the prognosis of DN under high glucose conditions (14). Glomerular hypertrophy, mesangial matrix hyperplasia, vacuoles and granular degeneration of renal tubular epithelial cells, renal tubular expansion, irregular thickening of basement membrane and small focal mononuclear macrophage infiltration in the tubular interstitium were observed in the STZ-induced diabetic rats in the present study. Twenty-four hour urinary protein increases and impaired renal function were also identified in these rats. The aforementioned results confirmed that the experimental model of DN was successful.

The phenotype of renal tubular epithelial cells changes as a result of a variety of pathological factors in the development of DN. The expression of epithelial marker antigens, including cytokeratin and cadherin, is lost (15). Mesenchymal cell marker, including α-SMA, vimentin and fibroblast specific protein, expression is initiated and extracellular matrix (ECM) is produced and secreted, indicating EMT (16). TGF-β_1_ is hypothesized to have a key role in this process (6,17). α-SMA expression is widely used for the detection of EMT, and its expression is restricted to renal vascular smooth muscle cells since there are almost no myofibroblasts in the normal kidney (18). When α-SMA expression is detected in the glomerular mesangial cells, renal tubular epithelial cells, renal interstitial fibroblasts and/or other inherent renal cells, their cell phenotype begins to transform from static to proliferative or secretory type cells, initiating ECM synthesis and secretion and inducing the development of myofibroblast properties (19). The results of the present study indicated that α-SMA expression confined to the renal tissue vascular smooth muscle cells in the normal control group; whereas α-SMA expression was significantly increased in the renal tubular interstitium, as well as on the walls of the blood vessels in the DM group. α-SMA expression was lower in the renal tubular interstitium of all the medicated groups compared with that of the DM group. Furthermore, expression was significantly lower in the combined therapy group than the single-drug treatment groups, suggesting that the EMT of renal tubule epithelium occurring following 12 weeks in the DM group, was partially inhibited by benazepril or MMF treatment alone, and combined treatment had synergistic inhibitory effects on EMT in diabetes.

ACEIs are known to improve diabetic glomerular hemodynamics, reduce glomerular hypertension and proteinuria by blocking the renin-angiotensin aldosterone system, as well as exerting renal protective effects independent of its anti-hypertensive effect (20). Previous studies have shown that angiotensin II (Ang II) has a significant role in the process of renal tubular interstitial fibrosis repair. Ang II stimulates renal tubular epithelial cell hypertrophy, induces TGF-β_1_ production and induces the differentiation of fibroblasts and renal tubular epithelial cells into myofibroblasts. ECM production is increased and degradation is decreased via the RAS system and Ang II type I receptors, which results in tubular interstitial fibrosis (21). Furthermore, Ang II, as an inflammatory factor, induces the activation and translocation of inflammation-initiated nuclear factor-κB in the early stages of DN (22), leading to the expression of cytokines and adhesion molecules, including intercellular adhesion molecule 1, monocyte chemotactic factor (MCP-1) and osteopontin, and the recruitment of large numbers of inflammatory cells infiltrating into the glomerular and renal tubular interstitium. In the present study, benazepril was used to block Ang II production, and renal hypertrophy, matrix accumulation, glomerular and renal interstitial fibrosis were alleviated following treatment, consistent with previous findings.

MMF, as a novel immune inhibitor, inhibits T and B lymphocyte proliferation, monocyte and mesangial cell proliferation, tubular interstitial mononuclear macrophage infiltration and the synthesis of multiple cytokines, including TGF-β_1_, MCP-1 and TNF-α (23). MMF has therefore been widely used in the treatment and prevention of transplant rejection, autoimmune diseases and primary glomerular disease. Roos *et al* (24) revealed that MMF was able to reduce the generation of α-SMA and type I collagen in cultured rat lung fibroblasts in a systemic sclerosis model, suggesting that MMF had direct anti-fibrotic effects. Whether MMF also has a direct anti-fibrosis role in DN remains to be elucidated.

In conclusion, the results of the present study demonstrated that MMF and benazepril have protective effects in a rat model of DN. Furthermore, combined treatment with MMF and benazepril had significantly greater protective effects than single drug treatments. The underlying mechanism may be associated with the inhibition of renal tubular epithelial cell transdifferentiation, but this hypothesis requires further investigation.
